# Sudden unexplained death in a patient with HIV and MDR-TB

**DOI:** 10.1186/1758-2652-13-S4-P198

**Published:** 2010-11-08

**Authors:** E Boxall, J Milne, E Peters, C Sykes

**Affiliations:** 1 Brownlee Centre, Department of Infectious Diseases, Glasgow, UK

## Purpose of the study

We report the case of a patient who presented with HIV and MDR-TB who died unexpectedly of presumed cardiac arrhythmia.

## Methods

A 25 year old Lithuanian man was admitted with symptoms consistent with TB. He had cavitation on CXR consistent with TB. He was HIV+ve with a CD4 count of 16. His CMV titre was low positive. Standard TB treatment was commenced on day 4. He started efavirenz and Truvada on day 12. On Day 18, his TB proved to be multi-drug resistant; his anti-tuberculous therapy was changed to IV amikacin, moxifloxacin, prothionamide, ethambutol, and pyrazinamide. Cycloserine and linezolid were added on day 21. Efavirenz was changed to darunavir/boosted ritonavir due to psychiatric side effects at day 28, and clofazamine and valganciclovir were added on day 35. Fluconazole was added due to oral candidiasis at day 45. Clinically, he improved over the subsequent few days, and his CD 4 was 121 by day 41.

He was found dead unexpectedly on the ward on day 52, and the presumed cause of death was cardiac arrhythmia. He was on low molecular weight heparin and had been well during the week prior to his death. There was no clinical suspicion of cardiac disease. Observations were stable the day of his death and on day 50 he had Hb 80g/dl WCC 1.51(neuts 0.78) CRP 78 and K+3.2 which were improved or similar to baseline. No post mortem was performed.

## Results

HIV is associated with cardiovascular complications, including ischaemic heart disease, cardiomyopathy and sudden death due to arrhythmia. It is also associated with high early mortality in MDR-TB. He was on moxifloxacin, ondansetron, ritonavir and fluconazole which are all known to prolong the QT interval. We suspect he had a fatal arrhythmia due to his medication, plus or minus HIV infection in combination with MDR-TB. Immune reconstitution is postulated to cause sudden death, but there was no evidence of cytokine storm in this case.

## Conclusion

We now plan to perform regular ECGs on patients on long-term moxifoxacin and exercise caution in using multiple agents which prolong the QT interval.

